# Anti-Inflammatory and Antinociceptive Activities of Bufalin in Rodents

**DOI:** 10.1155/2014/171839

**Published:** 2014-02-26

**Authors:** Lili Wen, Yang Huang, Xianbiao Xie, Wan Huang, Junqiang Yin, Wenqian Lin, Qiang Jia, Weian Zeng

**Affiliations:** ^1^Department of Anesthesiology, State Key Laboratory of Oncology in South China, Sun Yat-Sen University Cancer Center, 651 Dongfeng East Road, Guangzhou 510060, China; ^2^Department of Orthopaedic Oncology, First Affiliated Hospital of Sun Yat-Sen University, Guangzhou 510080, China; ^3^The Institute of Biology, Guizhou Academy of Sciences, Guiyang 550009, China

## Abstract

The aims of this study were to evaluate the anti-inflammatory and analgesic effects of bufalin, a major component of “Chan-su.” We used a carrageenan-induced paw edema model to assess the anti-inflammatory activity of this compound, and Western blot analysis detected NF-**κ**B signaling during this effect. The antinociceptive activities were evaluated by acetic acid-induced writhing, formalin, and hot-plate tests; open-field test investigated effects on the central nervous system. Our data showed that bufalin (0.3 and 0.6 mg/kg, i.p.) potently decreased carrageenan-induced paw edema. Bufalin down regulated the expression levels of nitric oxide synthase (iNOS), cyclooxygenase-2 (COX-2), interleukin-1**β** (IL-1**β**), interleukin-6 (IL-6), and tumor necrosis factor-**α** (TNF-**α**) during these treatments. Further studies demonstrated that bufalin significantly inhibited the activation of NF-**κ**B signaling. Bufalin also reduced acetic acid-induced writhing and the licking time in the formalin test and increased hot-plate reaction latencies. Naloxone pretreatment (2 mg/kg, i.p.) in the early phases of the formalin test and hot-plate test significantly attenuated the bufalin-induced antinociception effects, which suggests the involvement of the opioid system. A reduction in locomotion was not observed in the open-field test after bufalin administration. Taken together, bufalin treatment resulted in in vivo anti-inflammatory and analgesic effects, and bufalin may be a novel, potential drug for the treatment of inflammatory diseases.

## 1. Introduction

Bufalin is the main component extracted from toxins in “Chan-su,” which is known as toad venom [1]. Chan-su is a traditional Chinese medicine that is obtained from the skin and parotid venom glands of the Asiatic toad *Bufo gargarizans* Cantor. Chan-su has long been used as a therapeutic agent in China and other Asian countries because of its effects on numerous biological activities, such as cardiotonic, blood pressure stimulation, anti-inflammatory, anesthetic, and antineoplastic activities [2, 3]. Bufalin is the major digoxin-like component of Chan-su and may increase vasoconstriction, vascular resistance, and blood pressure through the inhibition of Na^+^/K^+^-ATPase activity [4]. This compound also exhibits a variety of biological effects, including anesthetic, respiratory excitation, inhibition of cell proliferation, induction of cell differentiation and apoptosis, disruption of the cell cycle, inhibition of angiogenesis, reversing multidrug resistance, and regulation of the immune response [5–15]. Accumulating evidence has revealed that bufalin also induces a number of cellular signaling events that are independent of Na^+^/K^+^-ATPase suppression [5–15].

Chan-su has been used in the treatment of inflammatory diseases, such as tonsillitis and sore throat, for thousands of years in China [3], but very little is known about the anti-inflammatory effects of bufalin. Recently, Ye et al. [16] demonstrated that bufalin inhibits the nuclear translocation of NF-*κ*B in response to TNF in vitro. Bufalin also modulated NF-*κ*B activity in cancer cells in our previous study [12]. NF-*κ*B is a central regulator of the inflammatory process and plays a critical role in inflammation. This molecule regulates the expression of a group of proinflammatory mediators, such as cyclooxygenase-2, inducible nitric oxide synthase, TNF, IL-1*β*, and IL-6 [17]. NF-*κ*B signaling is an optimal therapeutic target for the pathogenesis of inflammation. Therefore, the aim of the current study was to confirm the anti-inflammatory and analgesic effects of bufalin and determine the role of NF-*κ*B and the proinflammatory mediators COX-2, iNOS, TNF-*α*, IL-1*β*, and IL-6 to evaluate the potential of bufalin as an alternative drug in the treatment of inflammatory diseases.

## 2. Materials and Methods 

### 2.1. Drugs and Chemicals

Bufalin, indomethacin, carrageenan, morphine, acetic acid, formaldehyde, naloxone, and diazepam were purchased from Sigma-Aldrich (St. Louis, MO, USA). NF-*κ*B p65, I*κ*B*α*, and *β*-actin antibodies were purchased from Cell Signal Technologies (Danvers, MA, USA); iNOS, COX-2, TNF-*α*, IL-6, and IL-1*β* antibodies were purchased from Abcam (Abcam, Cambridge, MA, USA). The antibodies for *α*-tubulin and lamin A/C were obtained from Santa Cruz Biotechnology (Santa Cruz, CA, USA).

### 2.2. Animals

Male Sprague-Dawley rats (200–220 g) and male ICR mice (18–22 g) were purchased from the Experimental Animal Center of Guangdong Province, China. Animals were maintained in an air-conditioned room (21 ± 1°C and 50–60% humidity) with a controlled 12 h light-dark cycle. Standard food and water were provided ad libitum. All the studies were conducted in accordance with the guidelines of the Center of Experiment Animal of Sun Yat-sen University, and the ethics committee of the Center of Experiment Animal of Sun Yat-sen University approved all studies.

### 2.3. Carrageenan-Induced Paw Edema

The anti-inflammatory effect was based on the inhibition of carrageenan-induced hind paw edema. Bufalin (0.15, 0.3, and 0.6 mg/kg, i.p.), indomethacin (10 mg/kg, i.p.), and vehicle were administered intraperitoneally 30 min prior to carrageenan injection. Each rat received a subplantar injection of carrageenan (0.1 mL, 1% w/v in saline) in the right hind paw. A plethysmometer (Ugo Basile, Italy) measured the volume of paw edema before and at various times (1, 2, 4, and 6 h) after the carrageenan injection. The paw swelling ratio was calculated as a percentage increase from the paw volume measured prior to carrageenan injection. Rat paws were collected after the final assessment. Each hind paw was cut at the level of the calcaneus bone and used for Western blot analysis.

### 2.4. Preparation of Protein Extracts

Whole tissue protein extracts were prepared. Frozen tissues were homogenized in ice-cold lysis buffer, which consisted of 250 mM sucrose, 1 mM MgCl_2_, 1 mM DTT, 2.5 mM EDTA, 1 mM EGTA, 50 mM NaF, 10 lg/mL leupeptin, 1.25 lg/mL pepstatin, 2.5 lg/mL aprotin, 2 mM sodium pyrophosphate, 0.1 mM NaVO4, 0.5 mM PMSF, and protease inhibitor cocktail (Roche Diagnostic, Manheim, Germany). The samples were sonicated on ice and centrifuged at 15,000 ×g for 15 min at 4°C to isolate the supernatants. Protein concentrations were determined using the Bradford assay. The extracts were aliquoted and used for immunoblotting.

Nuclear extracts were prepared using a Nuclear Extract Kit (Active Motif, Carlsbad, CA, USA) as previously described [12]. Briefly, tissue was washed with 1 mL prechilled PBS with phosphatase inhibitors, lysed in 500 *μ*l hypotonic buffer, and centrifuged at 14,000 ×g for 30 s at 4°C. The supernatant was transferred to fresh 1.5 mL microtubes as the cytoplasmic fraction. The pellets were resuspended in 50 µl of complete lysis buffer and centrifuged at 14,000 ×g for 10 min at 4°C. The supernatants (nuclear fraction) were saved. Western blot analysis was used to determine the protein levels of NF-*κ*B p65 in the nucleus.

### 2.5. Western Blot Analysis

Protein extracts (50 *μ*g) from each sample were separated by SDS-PAGE and transferred to polyvinylidene difluoride membranes. The membranes were incubated with primary antibodies for 12–18 h at 4°C after three washes in Tris-buffered saline with Tween 20 (TBST) for 5 min, followed by incubation with secondary antibody for 1 h at room temperature. Protein bands were visualized on X-ray film using an enhanced chemiluminescence detection system. Densitometric analysis determined the amount of protein in each sample.

### 2.6. Acetic Acid-Induced Writhing Test

An acetic acid abdominal writhing test was performed in mice. Animals were placed separately into clear plastic cages for observation and to calculate abdominal writhing. Abdominal writhing was defined as an exaggerated extension of the abdomen combined with the outstretching of the hind limbs. Bufalin (0.15, 0.3, and 0.6 mg/kg, i.p.), morphine (5 mg/kg, i.p.), and vehicle were injected intraperitoneally 30 min before acetic acid (0.6% w/v, 10 mL/kg) administration. The number of writhing was calculated at a start time of 5 min after acetic acid injection and continued for 20 min. The antinociceptive effects are shown as the percent inhibition relative to the average number of writhing observed in the vehicle group.

### 2.7. Detection of TNF-*α* and IL-1*β* Produced by Peritoneal Cells Harvested from Peritoneal Cavities of Animals That Underwent Acetic Acid-Induced Writhing Tests

Mice were pretreated i.p. with vehicle or bufalin (0.6 mg/kg) 30 min before the i.p. administration of acetic acid (0.6% w/v, 10 mL/kg). The peritoneal cavities were then washed with saline (1 mL/cavity) after 15 min. The exudates were centrifuged at 300 g for 10 min. The peritoneal cells were resuspended in RPMI with 10% fetal calf serum and cultured in 96-well plates. After culturing for 12 h, the concentrations of TNF-*α* and IL-1*β* in the supernatants were detected by ELISA as previously described [18]. Absorbance was measured at 490 nm. The results were shown as the means ± S.E.M. for five animals.

### 2.8. Formalin Test

Mice were pretreated with an intraperitoneal injection of vehicle, 0.15, 0.3, or 0.6 mg/kg bufalin, or 5 mg/kg morphine (positive control) 30 min before the intraplantar injection of formalin (20 *μ*l, 2.5% in saline) solution into the right hind paw. The animals were placed immediately in a glass cylinder, and the time spent licking the injected paw was considered indicative of nociception. Responses were recorded from 0 to 5 min (first phase, neurogenic) and from 15 to 30 min (second phase, inflammatory) following formalin injection.

### 2.9. Hot-Plate Test

The hot plate was maintained at 55 ± 0.5°C. The mice were placed on the heated surface, and the time (in seconds) between placement and the licking of hind paws or jumping was recorded as the response latency. A 40-s cutoff time was used to minimize tissue damage. Animals presenting baseline latencies higher than 20 s were excluded. The hot-plate latency on the day of the experiment was measured at 0, 30, 60, and 120 min after the administration of bufalin (0.15, 0.3, and 0.6 mg/kg, i.p.), morphine (5 mg/kg, i.p.), or vehicle.

### 2.10. Assessment of Opioid System Involvement in Bufalin Antinociceptive Activity

Three groups of animals received naloxone (2 mg/kg, i.p., a nonselective opioid receptor antagonist) to evaluate the participation of the opioid system. These animals received vehicle, morphine (5 mg/kg, i.p.), or bufalin (0.6 mg/kg, i.p.) 15 min after naloxone administration. The other three groups received only vehicle, morphine (5 mg/kg, i.p.), or bufalin (0.6 mg/kg, i.p.). The formalin and hot-plate tests were subsequently performed.

### 2.11. Open-Field Test

The open-field test assessed mouse ambulatory behavior. Groups of mice were treated with vehicle, diazepam (1 mg/kg, i.p., as a reference drug), or bufalin (0.15, 0.3, and 0.6 mg/kg, i.p.) 30 min before the open-field test. Each animal was placed into the center of the open-field area and was allowed to freely ambulate for 5 min. The number of areas crossed with all paws was recorded.

### 2.12. Statistical Analysis

All data were expressed as the means ± standard error. Significant differences were assessed using an analysis of variance (ANOVA), followed by Tukey's post hoc test.* P* < 0.05 was considered statistically significant.

## 3. Results

### 3.1. Anti-Inflammatory Activity of Bufalin on Carrageenan-Induced Paw Edema in Rats

**Figure 1:**
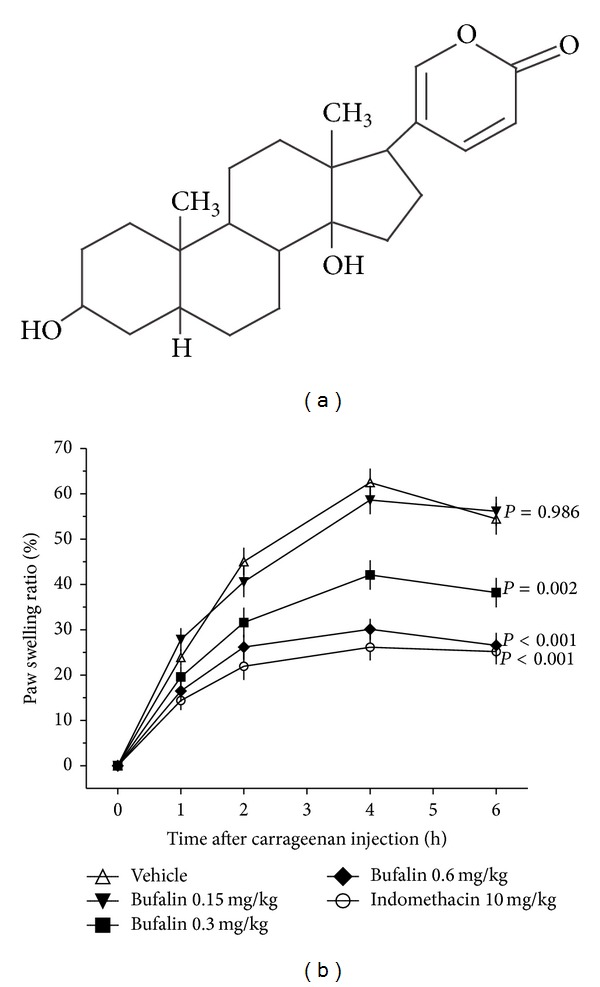
Effect of bufalin on carrageenan-induced rat paw edema. (a) Chemical structure of bufalin. (b) Bufalin at doses of 0.15, 0.3, and 0.6 mg/kg, the reference drug indomethacin at 10 mg/kg, and the vehicle were administered to rats 30 min before carrageenan injection. The percentage of increase in paw volume of the right hind paws of each rat at each time point was calculated. Data are expressed as the mean ± S.E.M. (*n* = 8), *P* value, compared with the vehicle group.

The carrageenan-induced paw edema model was employed to assess the anti-inflammatory activity of bufalin (see Figure 1(a) for the chemical structure) on acute inflammation in vivo. Carrageenan-induced intense paw edema reached a maximum level at 4 h after injection, and the edema decreased during the subsequent hour (Figure 1(b)). Significant reductions in paw volumes were observed in the test groups treated with either 0.3 or 0.6 mg/kg bufalin compared with the vehicle group following carrageenan injection (*P* < 0.01, *P* < 0.001, resp.). The lowest dose of bufalin (0.15 mg/kg) did not suppress paw edema compared with the vehicle group. The reference drug, indomethacin (10 mg/kg), significantly suppressed paw edema. The highest dose of bufalin (0.6 mg/kg) demonstrated a comparable level of inhibition to indomethacin (10 mg/kg) (*P* > 0.05). These initial results indicate that bufalin possesses strong anti-inflammatory activity in vivo in a dose-dependent manner in tissues with acute inflammation.

### 3.2. Bufalin Inhibited Inflammatory Signaling in the Carrageenan-Induced Paw Edema Model

**Figure 2:**
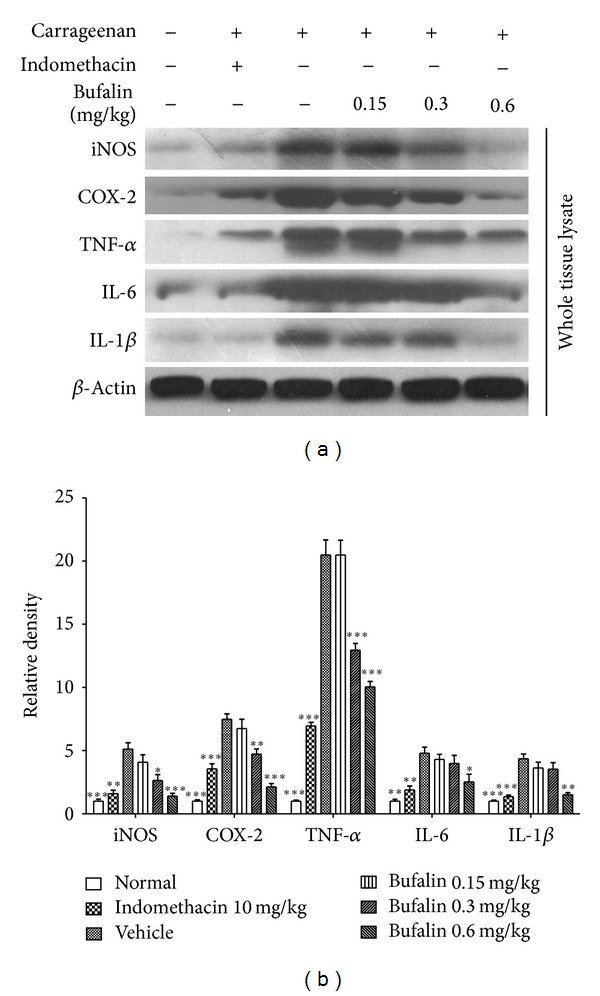
Bufalin inhibited proinflammatory mediators in the carrageenan-induced paw edema model. (a) Effect of bufalin on iNOS, COX-2, TNF-*α*, IL-1*β*, and IL-6 protein expressions in the carrageenan-induced paw edema model as detected using western blot. (b) The protein expression levels were analyzed by Image J.2x software. Data are expressed as the mean ± S.E.M. of 3 determinations, *P < 0.05, **P < 0.01, and ***P < 0.001, compared with the vehicle group.

Immunoblotting analysis examined inflammatory signaling to further identify the anti-inflammation activity of bufalin. Bufalin reduced the protein expression of iNOS and COX-2 in a dose-dependent manner in carrageenan-injected paw tissues (Figure 2). Treatment with 0.3 and 0.6 mg/kg bufalin reduced iNOS protein expression by approximately 48.37% and 72.61%, respectively, and COX-2 protein expression by 36.93% and 71.63%, respectively. No significant difference was found at 0.15 mg/kg. Indomethacin (10 mg/kg, i.p.) significantly inhibited iNOS and COX-2 protein expressions. The downstream proinflammatory cytokines TNF-*α*, IL-1*β*, and IL-6 were also detected using Western blot analysis (Figure 2). Carrageenan injection induced a remarkable increase in the expression of TNF-*α* and IL-1*β* and a mild increase in IL-6 expression. Bufalin attenuated the carrageenan-induced increase in expression of these cytokines in a dose-dependent manner. These data further demonstrate that bufalin inhibits inflammatory signaling in the context of acute inflammation in vivo in a dose-dependent manner.

### 3.3. Bufalin Interfered with Activation of NF-*κ*B Signaling in the Carrageenan-Induced Paw Edema Model

**Figure 3:**
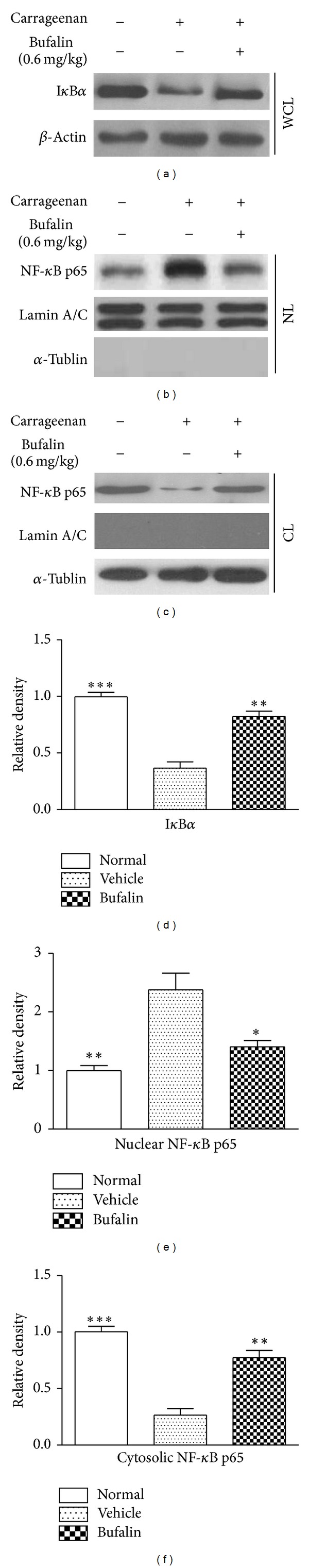
Bufalin interfered with activation of NF-*κ*B signaling in the carrageenan-induced paw edema model. In this experiment, the dose of bufalin was 0.6 mg/kg. (a) The level of I*κ*B*α* was detected by western blot. The nuclear (b) and cytosolic (c) NF-*κ*B p65 were monitored after 0.6 mg/kg bufalin injection by western blot. The levels of I*κ*B*α* (d), nuclear (e), and cytosolic (f) p65 were analyzed using Image J.2x software. Data are expressed as the mean ± S.E.M. of 3 determinations, *P < 0.05, **P < 0.01, and ***P < 0.001, compared with the vehicle group. WCL: whole cell lysate, NL: nuclear lysate, and CL: cytosolic lysate.

NF-*κ*B is a central molecule in the inflammatory cascade. To study whether the anti-inflammation effects of bufalin could be related to the inhibition of NF-*κ*B activation, the effects of bufalin on the protein level of I*κ*B*α* and NF-*κ*B translocation were examined. As shown in Figures 3(a) and 3(d), animals treated with carrageenan had a significant decrease in I*κ*B*α* levels; however, bufalin pretreatment could attenuate this effect. We also monitored the nuclear translocation of p65 in the presence or absence of bufalin. In nuclear fractions from carrageenan- and bufalin-treated animals, NF-*κ*B p65 was approximately 2.38- and 1.40-fold, respectively, compared with the control group (Figures 3(b) and 3(e)). The cytosolic level of NF-*κ*B p65 in the bufalin group was significantly higher than that in the carrageenan group (Figures 3(c) and 3(f)). These results suggest that bufalin administration could significantly inhibit the activation of NF-*κ*B by maintaining I*κ*B*α* levels and reducing the nuclear translocation of NF-*κ*B p65 after carrageenan injection.

### 3.4. Antinociceptive Effects of Bufalin

**Figure 4:**
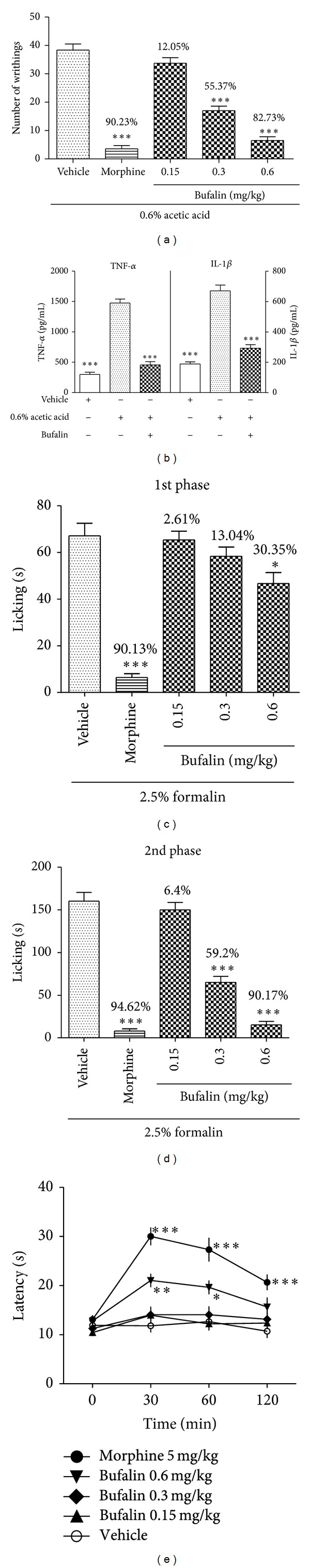
The antinociceptive effect of bufalin. (a) Bufalin (0.15, 0.3, or 0.6 mg/kg), the reference drug morphine (5 mg/kg), and the vehicle were administered to mice 30 min before a peritoneal injection of acetic acid. The numbers of abdominal writhes were recorded. The inhibition rate of the writhes for 20 min following acetic acid injection was calculated (*n* = 8). (b) ELISA was used to test TNF-*α* and IL-1*β* produced by peritoneal cells harvested from peritoneal cavity of animals that underwent acetic acid-induced writhing tests (*n* = 5). Effects of bufalin and morphine on the licking time of the formalin test in mice during the first (0–5 min) (c) and second phases (15–30 min) (d) (*n* = 8). (e) The hot plate latency was measured at 0, 30, 60, and 120 min after administration of bufalin, morphine, or vehicle (*n* = 8). Data are expressed as the mean ± S.E.M., *P < 0.05, **P < 0.01, and ***P < 0.001, compared with the vehicle group.

Three well-accepted pain models were used in our study to evaluate the role of bufalin in pain.  Figure 4(a) illustrates the number of the abdominal writhing stimulated by an intraperitoneal injection of acetic acid and the antinociceptive activity of bufalin in a mouse model. Bufalin treatment significantly inhibited the number of acetic acid-induced writhing in mice in a dose-dependent manner. The maximal inhibitory effect was observed at the highest bufalin dose (82.73%) (*P* < 0.001). The 0.3 mg/kg bufalin group also inhibited the nociceptive response by 55.37% compared with the vehicle group (*P* < 0.001). However, 0.15 mg/kg bufalin did not significantly suppress the number of writhing in mice. The morphine (5 mg/kg, i.p.) group exhibited a significant reduction in the number of writhing (90.23%). Furthermore, by ELISA, we detected TNF-*α* and IL-1*β* produced by peritoneal cells harvested from peritoneal cavities. The supernatants harvested from mice cavities stimulated with acetic acid showed significant increases in the amounts of TNF-*α* and IL-1*β* compared with the fluid harvested from mice injected with vehicle. Pretreatment with bufalin (0.6 mg/kg; i.p.) caused a significant decrease in TNF-*α* (−69%) and IL-1*β* (−56%) release compared with the acetic acid-stimulated group that was pretreated with vehicle (Figure 4(b)).

Bufalin (0.6 mg/kg, i.p.) treatment significantly reduced licking time by 30.35% during the neurogenic phase (0–5 min) of the formalin test compared with vehicle delivery (67.13 ± 5.36 s, *P* < 0.05) (Figure 4(c)). The administration of 0.3 and 0.6 mg/kg bufalin reduced vehicle licking time in the inflammatory phase (15–30 min) (160.25 ± 10.28 s) by 59.2% (*P* < 0.001) and 90.17% (*P* < 0.001), respectively. The lowest dose of bufalin (0.15 mg/kg, i.p.) did not reduce the paw licking time in either phase of the formalin test (Figure 4(d)). Morphine treatment significantly reduced licking time in both phases (90.13% in the first phase and 94.62% in the second phase).

Figure 4(e) shows the reaction latencies to the hot-plate tests recorded at 0, 30, 60, and 120 min after the administration of vehicle, bufalin, and morphine. The vehicle-treated group showed reaction latencies of 11.8 ± 1.34 s, 12.64 ± 1.31 s, and 10.69 ± 1.38 s at 30, 60, and 120 min after treatment, respectively. The lower doses of bufalin (0.15 and 0.3 mg/kg) did not increase the reaction latencies for any time point. Treatment with 0.6 mg/kg bufalin significantly increased the reaction latencies at 30 min (21.03 ± 1.37 s) and 60 min (19.63 ± 1.4 s) postadministration compared with the control (*P* < 0.01, *P* < 0.05, resp.). However, the latency at 120 min was not significantly altered (*P *> 0.05). Morphine increased the reaction latencies at 30 (30.01 ± 1.8 s), 60 (27.3 ± 2.43 s), and 120 (20.66 ± 1.59 s) min posttreatment.

### 3.5. Involvement of the Opioid Pathway in the Antinociceptive Effects of Bufalin

**Figure 5:**
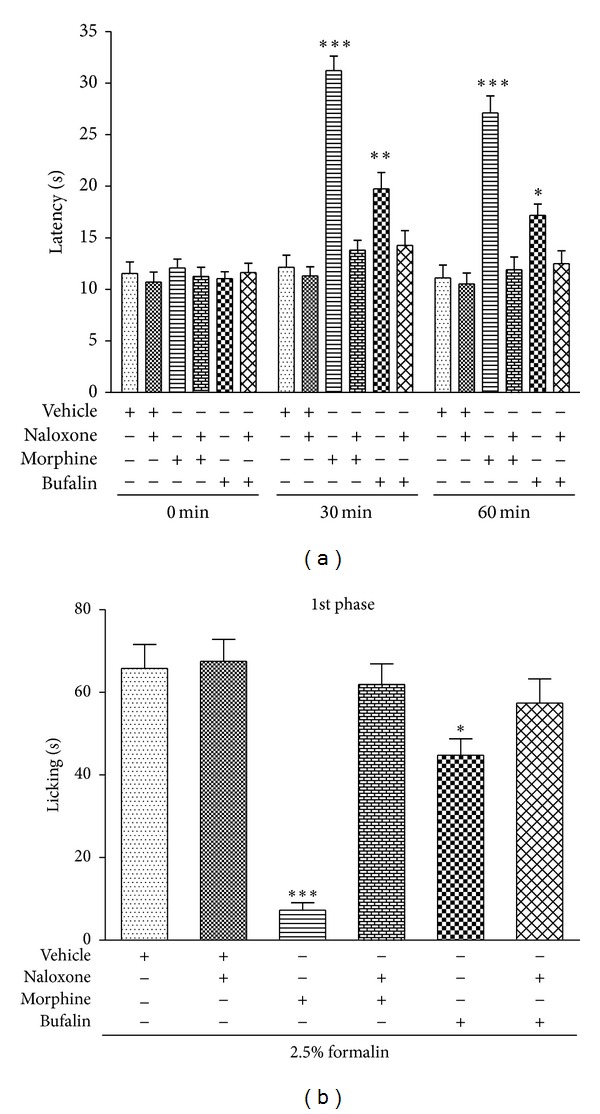
Pretreatment effect on mice with naloxone (2 mg/kg, i.p.) in the antinociception induced by bufalin (0.6 mg/kg, i.p.) in the hot-plate test (a) and the first phase of formalin test (b). Data are expressed as the mean ± S.E.M. (*n* = 8), *P < 0.05, **P < 0.01, and ***P < 0.001, compared with the vehicle group.

Naloxone (2 mg/kg, i.p.) blocked the antinociceptive effect of bufalin (0.6 mg/kg, i.p.) in the hot-plate test at 30 min (14.26 ± 1.43 s) and 60 (12.5 ± 1.24 s) min (Figure 5(a)). Naloxone pretreatment in mice inhibited the antinociceptive effect triggered by bufalin in phase 1 of the formalin test (Figure 5(b)). Naloxone significantly reversed the analgesic effect of morphine (5 mg/kg, i.p.) in both experiments (Figure 5).

### 3.6. Effect of Bufalin in the Open-Field Test

**Figure 6:**
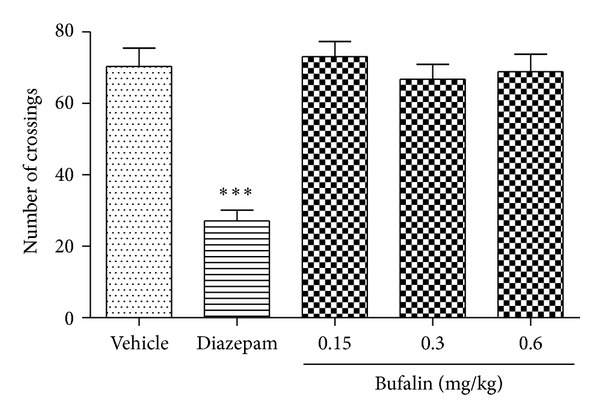
Effects of bufalin (0.15, 0.3, and 0.6 mg/kg, i.p.) or diazepam (1 mg/kg, i.p.) in the open- field test. The locomotion frequency was recorded. Data are expressed as the mean ± S.E.M. (*n* = 8), ***P< 0.001, compared with the vehicle group.

Bufalin (0.15, 0.3, or 0.6 mg/kg, i.p.) did not affect locomotion in mice. The number of areas crossed by all paws in the bufalin-treated groups was not significantly different from the vehicle-treated control group over a 5-minute period (*P* > 0.05) (Figure 6). In contrast, the reference drug diazepam (1 mg/kg, i.p.) significantly suppressed ambulatory behavior in mice.

## 4. Discussion

Bufalin, a main component of the traditional Chinese medicine Chan-su, exhibits numerous biological activities, such as cardiotonic effects, blood pressure stimulation, salt metabolism, anesthetic effects, antitumor activity, and immune response regulation. Several mechanisms of action have been proposed for bufalin, including the suppression of Na^+^/K^+^-ATPase [4, 6], Topo II [6], and ploy(ADP-ribose) polymerase 1 (PARP1) [7], the activation of AP-1 [8], Rac1 [9], cdc2 kinase, and casein kinase II [6], the induction of Tiam1 [9], and an increase of intracellular calcium concentrations [10]. Furthermore, bufalin down regulates apoptosis-related proteins, including Bcl-XL, Bcl-2, and Hsp27, and up regulates Bax and p21 [11, 12]. Bufalin also inhibits STAT3 [13] and AKT signaling pathways [14] and activates Fas- and mitochondria-mediated pathways [15].

The present study demonstrates that bufalin can exhibit a strong, dose-dependent anti-inflammatory effect on carrageenan-induced paw edema in rats, which is a commonly used model for the investigation of inflammation. The highest dose of bufalin demonstrated comparable anti-inflammatory activity to indomethacin. Bufalin alleviates ADR-induced proteinuria, which is closely related to inflammation and immune reactions [19]. The bufalin analogues ouabain and digoxin also possess strong anti-inflammatory effects [20, 21]. These studies prompted our investigation of the use of bufalin in inflammatory diseases. The exact mechanism of bufalin-induced anti-inflammation remains unclear.

Recently, Ye et al. reported that bufalin potently inhibited tumor necrosis factor (TNF) signaling by interfering with the nuclear translocation of NF-*κ*B in human 293T cells [16]. The present study found that bufalin significantly inhibited the activation of NF-*κ*B in vivo by maintaining I*κ*B*α* levels and reducing the nuclear translocation of NF-*κ*B p65. The downstream NF-*κ*B proinflammatory mediators iNOS, COX-2, TNF-*α*, IL-1*β*, and IL-6 were also reduced. NF-*κ*B is a central regulator of the inflammatory cascade and has played an extremely important role in the evolution and resolution phases of inflammation. NF-*κ*B is a “rapid-acting” primary transcription factor that acts as a “first responder” to inflammatory stimuli [17, 22, 23]. NF-*κ*B binds to the inhibitory protein I*κ*B0*α* and is located in the cytosol in the inactivated state. Inflammatory stimuli activate I*κ*B kinase (IKK). IKK phosphorylates I*κ*B*α*, which dissociates I*κ*B*α* from NF-*κ*B and leads to proteasomal degradation of I*κ*B*α*. Activated NF-*κ*B translocates to the nucleus and initiates the transcription of downstream inflammatory mediators, such as iNOS, COX-2, TNF-*α*, IL-1*β*, and IL-6 [17, 22, 23]. These mediators further activate NF-*κ*B through positive feedback. The targeting of NF-*κ*B and its downstream mediators is an important strategy for the suppression of inflammation [17]. Bufalin may inhibit activation of NF-*κ*B and reduce the production of its downstream proinflammatory mediators during acute inflammation.

Pain and edema are features of inflammation, and these fundamental and essential outcomes should be considered when evaluating potential anti-inflammatory compounds [23, 24]. Three murine pain models, acetic acid-induced writhing, formalin, and hot-plate tests, were employed in our study to evaluate the antinociceptive properties of bufalin. The acetic acid-induced writhing test is a typical model of inflammatory pain that is commonly used to screen new agents with peripheral analgesic and anti-inflammatory properties [18, 25]. Our data showed that bufalin significantly inhibited (in a dose-dependent manner) the number of acetic acid-induced writhing in mice. Bufalin doses of 0.3 and 0.6 mg/kg inhibited nociceptive responses by 55.37% and 82.73%, respectively. In the present study, we also demonstrated that bufalin could significantly reduce IL-1*β* and TNF-*α* levels released from resident peritoneal macrophages and mast cells in the peritoneal cavity. Therefore, the peripheral antinociceptive effect of bufalin may be related to the downregulation of the inflammatory mediators such as TNF-*α* and IL-1*β*, indicating that the antinociceptive effect of bufalin might correlate with its anti-inflammatory action [18, 26, 27].

A neurogenic and inflammatory pain model, the formalin test, was also used to further assess the antinociceptive properties of bufalin. Formalin administration elicits a biphasic behavioral response. The first phase (neurogenic phase) occurs during the first 5 min after formalin injection, and the behavioral effects are related to the direct chemical stimulation of nociceptors. The second phase (inflammatory phase) occurs during the 15th and 30th minutes after formalin injection, and this phase involves inflammatory pain that is induced by a combination of stimuli, including inflammation of peripheral tissues and mechanisms of central sensitization [25, 28]. Centrally acting drugs, such as opioids, inhibit both phases equally, but peripherally acting drugs, such as NSAIDs and corticosteroids, only inhibit the second phase [28]. Our results indicated that 0.3 mg/kg bufalin only reduced the pain response during the inflammatory phase of the formalin test, which suggests that the antinociceptive effect was specific to inflammatory pain [26, 27, 29]. On the other hand, the high dose bufalin (0.6 mg/kg) inhibited both phases of the formalin test, but its effect was more pronounced in the inflammatory phase. This result suggested that a high dose of bufalin also modulates acute neurogenic pain, which is sensitive to drugs with a central action [26, 27, 30].

We further examined the effects of bufalin using the hot-plate test to distinguish between central and peripheral antinociceptive effects. This test evaluates a possible central action in which opioid agents exert their analgesic effects via supraspinal and spinal receptors [31]. The highest dose of bufalin (0.6 mg/kg) increased the hot-plate response latencies at the 30 and 60 min posttreatment timepoints in our study. These results indicate that high-dose bufalin possesses central antinociceptive activity in murine pain models. These data also corroborate our previous studies on the antinociceptive activity of ouabain, another cardiac glycoside [32, 33]. We examined our hypothesis regarding the central antinociceptive activity of bufalin by using the hot-plate test and the first phase of the formalin test in animals pretreated with or without naloxone (an opioid-receptor antagonist). Interestingly, naloxone inhibited the antinociceptive effect of bufalin, which suggests that bufalin possesses an antinociceptive action that may be partially mediated by the activation of the opioid system [20, 26, 27].

Moreover, the open-field test was performed to exclude false positives, such as sedative effects on the animal behavior, in the nociceptive tests [26, 34]. We found that bufalin did not alter locomotor activity in the doses that yielded significant antinociception. These results indicate that the analgesic activity of bufalin is not related to sedative effects or motor disabilities [26, 34].

## 5. Conclusions

This study provides evidence that bufalin possesses strong in vivo anti-inflammatory activity, which may involve reduced activation of NF-*κ*B and the inhibition of downstream proinflammatory mediators. Bufalin has a potent antinociceptive effect that may be mediated through its anti-inflammatory action and activation of the opioid system. These results support bufalin as a novel potential therapeutic agent for the alleviation of inflammation and inflammatory pain.

## References

[1] Krenn L, Kopp B (1998). Bufadienolides from animal and plant sources. *Phytochemistry*.

[2] Qi F, Li A, Inagaki Y (2011). Antitumor activity of extracts and compounds from the skin of the toad Bufo bufo gargarizans Cantor. *International Immunopharmacology*.

[3] Chen KK, Kovaríková A (1967). Pharmacology and toxicology of toad venom. *Journal of Pharmaceutical Sciences*.

[4] Wang Z, Wen J, Zhang J, Ye M, Guo D (2004). Simultaneous determination of four bufadienolides in human liver by high-performance liquid chromatography. *Biomedical Chromatography*.

[5] Xie CM, Liu XY, Yu S (2013). Cardiac glycosides block cancer growth through HIF-1*α* and NF-*κ*B-mediated Plk1. *Carcinogenesis*.

[6] Numazawa S, Shinoki M, Ito H, Yoshida T, Kuroiwa Y (1994). Involvement of Na^+^,K^+^-ATPase inhibition in K562 cell differentiation induced by bufalin. *Journal of Cellular Physiology*.

[7] Huang H, Cao Y, Wei W (2013). Targeting poly (ADP-ribose) polymerase partially contributes to bufalin-induced cell death in multiple myeloma cells. *PLoS ONE*.

[8] Watabe M, Ito K, Masuda Y, Nakajo S, Nakaya K (1998). Activation of AP-1 is required for bufalin-induced apoptosis in human leukemia U937 cells. *Oncogene*.

[9] Kawazoe N, Watabe M, Masuda Y, Nakajo S, Nakaya K (1999). Tiam1 is involved in the regulation of bufalin-induced apoptosis in human leukemia cells. *Oncogene*.

[10] Yeh J, Huang WJ, Kan S, Wang PS (2003). Effects of bufalin and cinobufagin on the proliferation of androgen dependent and independent prostate cancer cells. *Prostate*.

[11] Yin J, Shen J, Su W (2007). Bufalin induces apoptosis in human osteosarcoma U-2OS and U-2OS methotrexate300-resistant cell lines. *Acta Pharmacologica Sinica*.

[12] Xie XB, Yin JQ, Wen LL (2012). Critical role of heat shock protein 27 in bufalin-induced apoptosis in human osteosarcomas: a proteomic-based research. *PLoS ONE*.

[13] Dong Y, Yin S, Li J, Jiang C, Ye M, Hu H (2011). Bufadienolide compounds sensitize human breast cancer cells to TRAIL-induced apoptosis via inhibition of STAT3/Mcl-1 pathway. *Apoptosis*.

[14] Li D, Qu X, Hou K (2009). PI3K/Akt is involved in bufalin-induced apoptosis in gastric cancer cells. *Anti-Cancer Drugs*.

[15] Qi F, Inagaki Y, Gao B (2011). Bufalin and cinobufagin induce apoptosis of human hepatocellular carcinoma cells via Fas- and mitochondria-mediated pathways. *Cancer Science*.

[16] Ye J, Chen S, Maniatis T (2011). Cardiac glycosides are potent inhibitors of interferon-*β* gene expression. *Nature Chemical Biology*.

[17] Rahman A, Fazal F (2011). Blocking NF-*κ*B: an inflammatory issue. *Proceedings of the American Thoracic Society*.

[18] Ribeiro RA, Vale ML, Thomazzi SM (2000). Involvement of resident macrophages and mast cells in the writhing nociceptive response induced by zymosan and acetic acid in mice. *European Journal of Pharmacology*.

[19] Zheng J, Gong J, Zhang A (2012). Attenuation of glomerular filtration barrier damage in adriamycin-induced nephropathic rats with bufalin: an antiproteinuric agent. *Journal of Steroid Biochemistry and Molecular Biology*.

[20] Rodrigues-Mascarenhas S, de Vasconcelos DIB, Leite JA (2011). Anti-inflammatory and antinociceptive activity of ouabain in mice. *Mediators of Inflammation*.

[21] Ihenetu K, Espinosa R, de Leon R, Planas G, Perez-Pinero A, Waldbeser L (2008). Digoxin and digoxin-like immunoreactive factors (DLIF) modulate the release of pro-inflammatory cytokines. *Inflammation Research*.

[22] Vallabhapurapu S, Karin M (2009). Regulation and function of NF-*κ*B transcription factors in the immune system. *Annual Review of Immunology*.

[23] Nathan C (2002). Points of control in inflammation. *Nature*.

[24] Morris CJ (2003). Carrageenan-induced paw edema in the rat and mouse. *Methods in Molecular Biology*.

[25] Le Bars D, Gozariu M, Cadden SW (2001). Animal models of nociception. *Pharmacological Reviews*.

[26] Costa EA, Lino RC, Gomes MN (2013). Anti-inflammatory and antinociceptive activities of LQFM002—a 4-nerolidylcatechol derivative. *Life Sciences*.

[27] Rodrigues JA, Vanderlei ES, Silva LM (2012). Antinociceptive and anti-inflammatory activities of a sulfated polysaccharide isolated from the green seaweed Caulerpa cupressoides. *Pharmacological Reports*.

[28] Shibata M, Ohkubo T, Takahashi H, Inoki R (1989). Modified formalin test: characteristic biphasic pain response. *Pain*.

[29] Munro G (2007). Dopamine D(1) and D(2) receptor agonism enhances antinociception mediated by the serotonin and noradrenaline reuptake inhibitor duloxetine in the rat formalin test. *European Journal of Pharmacology*.

[30] Nascimento MVM, Galdino PM, Florentino IF (2011). Antinociceptive effect of Lafoensia pacari A. St.-Hil. independent of anti-inflammatory activity of ellagic acid. *Journal of Natural Medicines*.

[31] Nemirovsky A, Chen L, Zelman V, Jurna I (2001). The antinociceptive effect of the combination of spinal morphine with systemic morphine or buprenorphine. *Anesthesia and Analgesia*.

[32] Zeng W, Chen X, Dohi S (2007). Antinociceptive synergistic interaction between clonidine and ouabain on thermal nociceptive tests in the rat. *Journal of Pain*.

[33] Zeng W, Dohi S, Shimonaka H, Asano T (1999). Spinal antinociceptive action of Na^+^-K^+^ pump inhibitor ouabain and its interaction with morphine and lidocaine in rats. *Anesthesiology*.

[34] Walsh RN, Cummins RA (1976). The open-field test: a critical review. *Psychological Bulletin*.

